# ZBTB34 is a hepatocellular carcinoma-associated protein with a monopartite nuclear localization signal

**DOI:** 10.18632/aging.204987

**Published:** 2023-08-30

**Authors:** Zheng Liu, Di Jin, Xinran Wei, Yue Gao, Xiaodie Gao, Xia Li, Xiujuan Wang, Pingying Wei, Tao Liu

**Affiliations:** 1 College of Medical Laboratory Science, Guilin Medical University, Guilin 541004, Guangxi, China; 2 Guihang Guiyang Hospital Affiliated to Zunyi Medical University, Guiyang 550027, Guizhou, China; 3 Clinical Laboratory, Hospital Affiliated to Guilin Medical University, Guilin 541001, Guangxi, China

**Keywords:** ZBTB34, nuclear localization signal, hepatocellular carcinoma, importin α

## Abstract

ZBTB34 is a novel zinc finger protein with an unknown function. In this study, the gene expression and survival prognosis of ZBTB34 were analyzed across tumors based on the TCGA datasets. According to the bioinformatics analysis and qPCR results, liver hepatocellular carcinomas exhibit a high level of ZBTB34 expression. Additionally, the experiment supported the bioinformatics analysis findings that ZBTB34 expression was regulated by miR-125b-5p and that ZBTB34 affected ZBTB10, POLR1B, and AUH expression in HepG2 cells. Biological software analysis further revealed that ZBTB34 contains a monopartite nuclear localization signal (NLS). Arginine and lysine inside the putative NLS were substituted using the alanine-scanning mutagenesis method. The findings showed that the ability of ZBTB34 to enter the nucleus was abolished by the alanine substitution of the sequence ^320^RGGRARQKRA^329^ and the mutation of Lys^327^ and Arg^328^ residues. ZBTB34 was co-immunoprecipitated with importin α1, importin α3, importin α4, and importin β1, according to the results of the co-immunoprecipitation assay. In conclusion, ZBTB34 is a hepatocellular carcinoma-associated protein with a monopartite NLS. The nuclear import of ZBTB34 is mediated by importin α1, importin α3, importin α4, and importin β1. ZBTB34 performs its biological functions via a putative miR-125b-5p/ZBTB34/(ZBTB10, POLR1B, and AUH) signaling axis in HepG2 cells.

## INTRODUCTION

Zinc finger proteins (ZFPs) are a superfamily of transcription factors that contain at least one zinc finger domain capable of binding specific DNA sequences and thus regulating DNA expression levels [1]. By identifying certain DNA sequences, the diverse combinations and functions of Zinc finger (ZnF) motifs enable ZFPs versatile in biological processes such as development, metabolism, and apoptosis [2]. Due to the multifunctional character of ZnF motifs, ZFPs are closely related to the progression and malignancy of cancer, being potential indicators of patient prognosis and survival [3]. ZFPs enter the nucleus, typically via the specific interaction of the importin α and importin β with its nuclear localization signal (NLS) [4]. NLS is an amino acid sequence within the primary structure of cargo proteins that target cargo proteins in the nucleus [5]. In general, importin α firstly binds to a specific class of NLS motifs, and then escorts the cargo into the nucleus in a complex with importin β. In the nucleus, ZFPs bind to cognate DNA site to regulate the expression of target genes.

Zinc finger protein 34 (ZBTB34) is a novel ZFP with 504 amino acids. Human ZBTB34 gene is located at Chromosome 9q33.3 with 1 exon. Since it was discovered in 2006, there are only eight reports on ZBTB34 in the PubMed database [6]. ZBTB34 is a transcriptional repressor that responds to reactive oxygen species-induced cell death and involved in the growth of colorectal cancer, according to recent studies [7, 8]. Our earlier research found that ZBTB34 is highly expressed in mouse embryonic stem cells, which causes telomere lengthening [9]. The mechanism is that ZBTB34 binds to telomere DNA via its third ZnF, competing for binding sites with Pot1b. Bioinformatics has produced promising results in analyzing gene functions in recent years [10]. In this study, we used bioinformatics tools to analyze the putative function of ZBTB34 and then verified the findings using various experiments. This approach could understand the biological functions of ZBTB34 quickly and accurately.

## RESULTS

### Data from gene expression analysis

**Figure 1:**
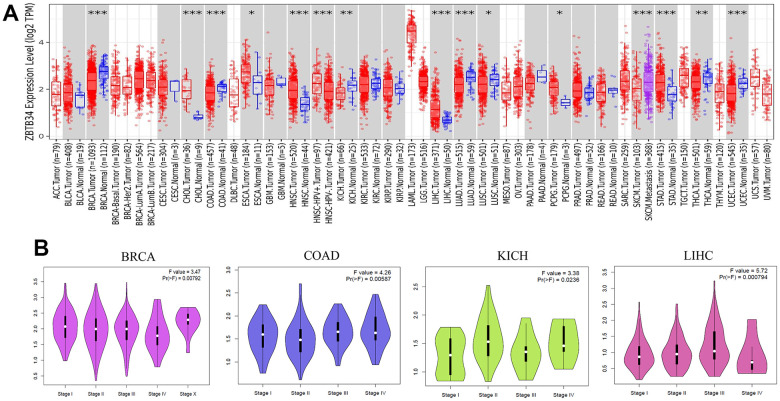
**The expression level of ZBTB34 gene in various tumor types and pathological stages.** (**A**) The expression status of the ZBTB34 gene in various cancers was analyzed through TIMER2 analysis. *P<0.05; **P<0.01; ***P<0.001. (**B**) Using TCGA data, the expression levels of the ZBTB34 gene were analyzed according to the four main pathological stages (stage I, stage II, stage III, and stage IV). Log2 (TPM+1) was applied for the log-scale.

The tumor immune estimation resource version 2 (TIMER2) web server offers comprehensive analysis and visualization functions for tumor-infiltrating immune cells across a wide range of cancer types [11]. We applied the TIMER2 approach to analyze the expression status of ZBTB34 across various cancer types. In contrast to the corresponding control tissues, ZBTB34 is expressed at significantly higher levels in the tumor tissues of cholangiocarcinoma (CHOL) (*P*<0.001), esophageal carcinoma (ESCA) (*P*<0.05), head and neck squamous cell carcinoma (HNSC) (*P*<0.001), liver hepatocellular carcinoma (LIHC or HCC) (*P*<0.001), pheochromocytoma and paraganglioma (PCPG) (*P*<0.05), and stomach adenocarcinoma (STAD) (*P*<0.001), as shown in Figure 1A. However, the expression of ZBTB34 in the tumor tissues of BRCA (Breast invasive carcinoma) (*P*<0.001), COAD (Colon adenocarcinoma) (*P*<0.001), KICH (Kidney chromophobe) (*P*<0.01), LUAD (Lung adenocarcinoma) (*P*<0.001), LUSC (Lung squamous cell carcinoma) (*P*<0.05), SKCM (Skin Cutaneous Melanoma) (*P*<0.001), THCA (Thyroid carcinoma) (*P*<0.01), and UCEC (Uterine Corpus Endometrial Carcinoma) (*P*<0.001) is significantly decreased in comparison with the control tissues (Figure 1A).

Next, we examined the correlation between ZBTB34 expression and the pathological stages of cancers using the “Pathological Stage Plot” module of Gene Expression Profiling Interactive Analysis version 2 (GEPIA2). The GEPIA web server has been a valuable and widely cited resource for gene expression analysis based on tumor and normal samples from The Cancer Genome Atlas (TCGA) and the GTEx databases [12]. Among these differentially expressed tumors, pathological stages of BRCA (F value=3.47; Pr=0.00792), COAD (F value=4.26; Pr=0.00587), KICH (F value=3.38; Pr=0.0236), and LIHC (F value=5.72; Pr=0.000794) correlated with ZBTB34 (Figure 1B). The F value is the difference between the residual (error) mean square and the model mean square [13]. It is used to determine whether the model has a statistically significant capacity for prediction as a whole. Under the null hypothesis that the model is not predictive, Pr (>F value) is the probability of finding an F value as extreme or more extreme. A value of Pr (>F) less than 0.05 means I reject the reduced model. Based on the study presented above, we surmise that the biological function of ZBTB34 is associated with BRCA, COAD, KICH, and LIHC.

### Data from survival analysis

**Figure 2:**
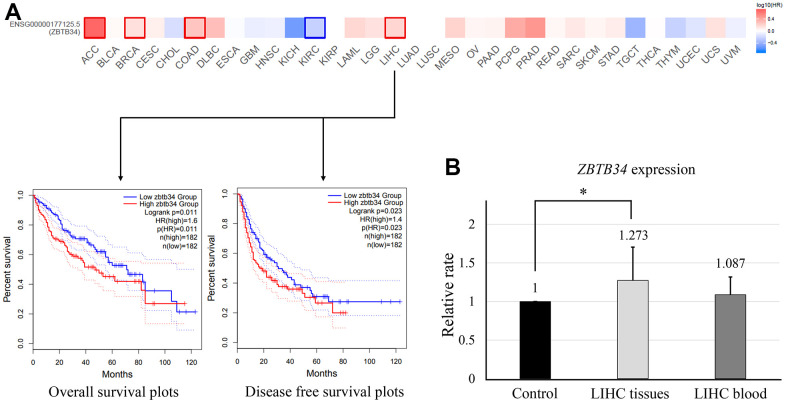
**Correlation between ZBTB34 gene expression and survival prognosis of cancers.** (**A**) The GEPIA2 tool was used to analyze the overall survival of various tumors in the TCGA data and ZBTB34 gene expression. (**B**) qPCR analyzes ZBTB34 expression in LIHC tissue and blood. ZBTB34 expression as fold changes compared to control values normalized to 1.

According to the ZBTB34 expression levels, we divided the cancer cases into high-expression and low-expression groups. Overall survival (OS) was measured from the date of the study, until the date of death or date last known alive [14]. Disease-free survival (DFS) is defined as the time from randomization to recurrence of tumor or death, and it is typically used in the adjuvant treatment setting [14]. Highly expressed ZBTB34 was associated with a poor prognosis of overall survival for adenoid cystic carcinoma (ACC) (*P*=0.00068), BRAC (*P*=0.038), COAD (*P*=0.013), and LIHC (*P*=0.011), as seen in the survival map of all tumors in Figure 2A. Lowly expressed ZBTB34 was associated with the poor prognosis of kidney renal clear cell carcinoma (KIRC) (*P*=0.00048). The correlation between ZBTB34 expression and LIHC in these five tumor types is consistent with the finding of gene expression analysis (Figure 1A). It indicates that the relationship between LIHC and ZBTB34 is supported by the evidence from bioinformatics analysis. The altered expression of ZBTB34 was also correlated with LIHC survival (Figure 2A). We then decided to conduct additional research to explore the relationship between ZBTB34 and LIHC. We collected blood samples from 21 Chinese LICH patients and 11 healthy individuals. We also collected cancer tissues and paracancerous tissues from 16 Chinese LIHC patients. Since ZFPs generally are housekeeping transcription factors that are constitutively expressed in cells and are essential for basic cellular functions, ethnicity shouldn’t be a significant factor affecting the analysis results [15]. The qPCR results showed that ZBTB34 expression was significantly higher in LICH tissue (*P*=0.010) compared to control, but not in peripheral blood (*P*=0.114) (Figure 2B). It implies that ZBTB34 is a putative oncogene that contributes to the development of LIHC.

### miR-125b-5p/ZBTB34/ (ZBTB10, POLR1B, and AUH) signaling axis

**Figure 3:**
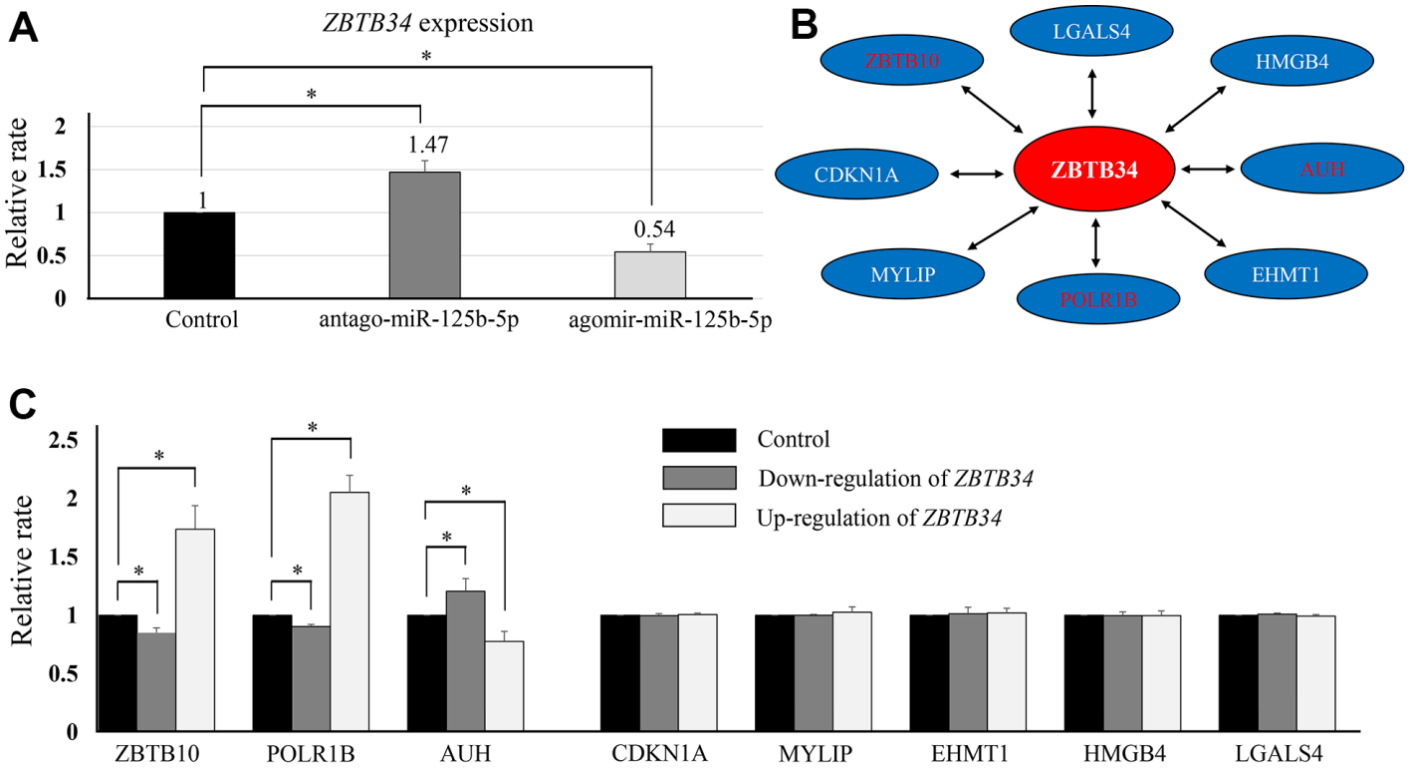
**The upstream and downstream regulators of ZBTB34.** (**A**) ZBTB34 was identified as the target gene of miR-125b-5p using the online software TargetScan. qPCR analysis showed ZBTB34 expression was lower in the ago-miR-125b-5p group and higher in the antago-miR-125b-5p group. ZBTB34 expression is expressed as a fold change compared to control values normalized to 1. (**B**) We also used the online software UCSC (http://genome.ucsc.edu/) and STRING (https://cn.string-db.org/) to predict the putative genes that interact with ZBTB34. A total of eight genes were predicted and selected for further study. (**C**) ZBTB34 increased ZBTB10 and POLR1B expression while decreasing AUH expression. Fold changes in gene expression compared to control values normalized to 1.

TargetScan software (https://www.targetscan.org) is a conservative program that predicts miRNA gene target sites by identifying 7mer and 8mer sequences that match the seed sequence for the targeting miRNA [16]. TargetScan software discovered miR-125b-5p regulating ZBTB34 expression. Using qPCR analysis, it was found that ZBTB34 expression was lower in the ago-miR--125b-5p group and higher in the antago-miR-125b-5p group (Figure 3A). Additionally, we predicted the putative ZBTB34-interacting proteins using the web tools UCSC (http://genome.ucsc.edu/) and STRING (https://cn.string-db.org/). The UCSC (UCSC) Genome Browser is an online graphical viewer for genomes, a genome browser, hosted by the University of California, Santa Cruz [17]. STRING (Search Tool for the Retrieval of Interacting Genes/Proteins) is a biological database and web resource of known and predicted protein-protein interactions [18]. A total of 8 genes were identified to further analyze (Figure 3B). ZBTB34 increased the expression of ZBTB10 and POLR1B while decreasing the expression of AUH in HepG2 (Figure 3C).

### ZBTB34 has a putative monopartite NLS

**Figure 4:**
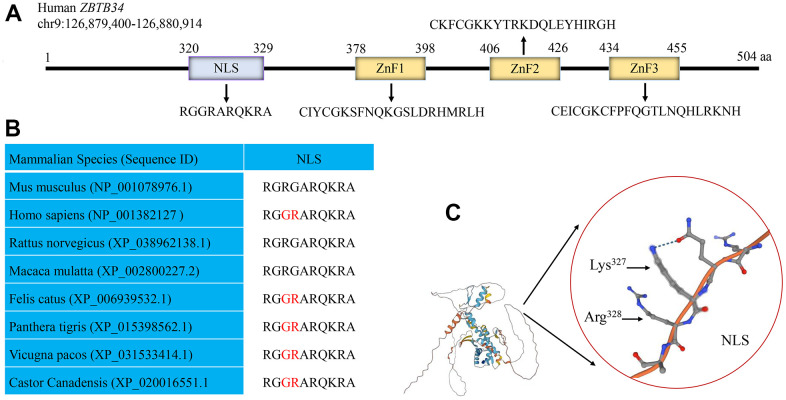
**Human ZBTB34 gene.** (**A**) Human ZBTB34 is located on chromosome 9 and contains one NLS and three ZnFs. (**B**) The alignment of the amino acid sequences within NLS among different mammalian species. (**C**) The position of NLS in ZBTB34 was determined using the AlphaFold proteins structure database. The accession number is indicated in parentheses. Non-conserved amino acid residues are highlighted in red. The red circle represents the position of NLS of ZBTB34, Lys^327^ and Arg^328^ of NLS.

NLStradamus is a hidden Markov model that accurately predicts localization signal sites [19]. The position weight matrices (PWM) predict DNA binding sites for Cys2His2 Zinc Finger Proteins [20]. By using the NLStradamus and the batch PWM software, a putative NLS sequence (^320^RGGRARQKRA^329^) and three ZnF domains were discovered within ZBTB34 (Figure 4A). The alignment of the amino acid sequences of *Mus musculus*, *Homo sapiens*, *Rattus norvegicus*, *Macaca mulatta*, *Felis catus*, *Panthera tigris*, *Vicugna pacos*, and *Castor canadensis* showed that the amino acids of NLS were highly conserved (Figure 4B). AlphaFold is an automated intelligence system developed by DeepMind that makes accurate predictions of a protein’s structure based on its amino-acid sequence [21]. Using the AlphaFold proteins structure database, the 3D structure of NLS on the ZBTB34 (UniProt: A0A0C4DFQ2) was identified (Figure 4C).

### ZBTB34 is a nuclear protein with a functional NLS

**Figure 5:**
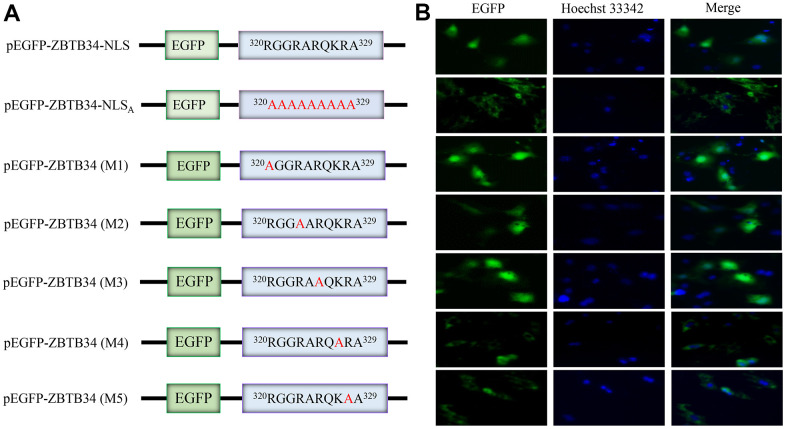
**ZBTB34 is a nuclear protein with a functional monopartite NLS.** (**A**) ZBTB34 cDNA was inserted into pEGFP-N1 to create pEGFP-ZBTB34. The amino acids in NLS were replaced with Ala to create pEGFP-ZBTB34-NLS_A_. pEGFP-ZBTB34 (M1), pEGFP-ZBTB34 (M2), pEGFP-ZBTB34 (M3), pEGFP-ZBTB34 (M4), and pEGFP-ZBTB34 (M5) were created by replacing the Lys or Arg motifs with Ala, respectively. (**B**) The nuclear location of EGFP-ZBTB34 fusion proteins, the EGFP-ZBTB34-NLS_A_ fusion protein, M1, M2, M3, M4, and M5 was analyzed using fluorescence microscopy. DNA stained with Hoechst appears blue.

ZBTB34 accumulated in the nuclei of the transfected cells when the pEGFP-ZBTB34 was transfected into HepG2 cells, demonstrating that ZBTB34 is a nuclear protein (Figure 5A). Alanine-scanning mutagenesis is a straightforward and widely used method for determining the catalytic or functional role of protein residues [22]. The alanine scanning method takes advantage of the fact that most canonical amino acids can be exchanged with Ala by point mutations, while the secondary structure of mutated protein remains intact, as Ala mimics the secondary structure preferences of the majority of the encoded or canonical amino acids. The pEGFP-ZBTB34-NLS_A_ (Ala substitution of the NLS) was constructed in order to experimentally validate the NLS sequence (^320^RGGRARQKRA^329^) of ZBTB34 (Figure 5B). After transfecting the pEGFP-ZBTB34-NLS_A_ into HepG2 cells, the green fluorescence emitted by EGFP was seen from the cytoplasm but not from the nucleus (Figure 5B). The loss of the NLS sequence caused a diffusion of ZBTB34 into the cytoplasm, in other words, abolished its nuclear entry ability.

### The Lys^327^ and Arg^328^ are critical for the nuclear import of ZBTB34

To further identify the key functional amino acids within NLS sequence, alanine-scanning mutagenesis assays were performed again. The basic residues Arg or Lys were mutated into Ala to generate mutants (M1, M2, M3, M4, and M5), respectively (Figure 5A). M1, M2 and M3 were localized in the nuclear of HepG2 cells, but the M4 and M5 were abolished in the nuclear accumulation (Figure 5B). According to these findings, Lys^327^ and Arg^328^ appear to be the crucial amino acid residues in responsible for importing nuclear localization of ZBTB34 and maintaining the function of NLS of ZBTB34. Furthermore, if ZBTB34 cannot enter the nucleus, it cannot perform its biological functions.

### Importins α1, α3, and α4 mediate nuclear import of ZBTB34

**Figure 6:**
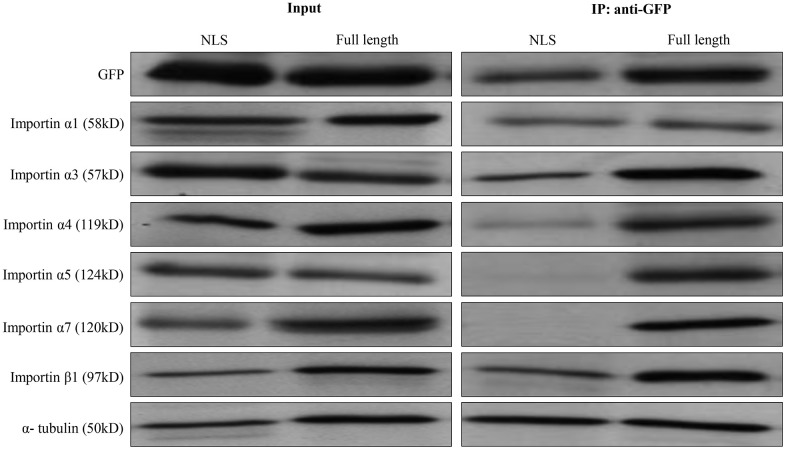
**The identification of importin α/β mediated ZBTB34.** Co-immunoprecipitation with Western blot (anti-GFP antibody) technique was used to analyze the NLS of ZBTB34 bind to importin α1, importin α3, importin α4, importin α5, importin α7, or importin β1 in HepG2 cells. The results show pEGFP-ZBTB34-NLS is co-immunoprecipitated with importin α1, importin α3, importin α4, importin β1, but not with importin α5 or importin α7. The input was used as a control for protein detection.

NLS-containing protein is recognized by one or more importin α members, which in turn is recognized by the importin β to help cargo protein enter the nuclear. We performed co-immunoprecipitation with Western blotting experiment and found that importins α1, importin α3, importin α4, and importin β1 were co-immunoprecipitated with ZBTB34 (Figure 6). These results indicated that nuclear import of ZBTB34 in HepG2 cells was mediated by importin α1, importin α3, importin α4, and importin β1.

## DISCUSSION

ZBTB34 is a novel ZFP with an unknown function. In this study, we analyzed ZBTB34 expression in a total of 32 different tumors based on the data of the TCGA database which is one of the largest publicly available and high-quality data sources for cancer-genomic studies [23]. The results showed that ZBTB34 was highly expressed in CHOL, ESCA, HNSC, LIHC, PCPG, and STAD. It is lowly expressed in BRCA, COAD, KICH, LUAD, LUSC, SKCM, THCA, and UCEC. Next, we discovered a correlation between ZBTB34 expression and the pathogenic stages of BRCA, COAD, KICH, and LIHC. Furthermore, lowly expressed ZBTB34 was associated with the poor prognosis of KIRC whereas highly expressed ZBTB34 was associated with the poor prognosis of ACC, BRAC, COAD, and LIHC malignancies. Finding common outcomes from bioinformatics analysis is crucial for understanding the function of ZBTB34 because bioinformatics analysis yields a variety of results. We draw the conclusion that BRAC, COAD, and LIHC are related to the highly expressed ZBTB34 based on the findings of the above analysis. ZBTB34 was, however, lowly expressed in BRCA and COAD, according to TCGA database analysis. As a consequence, only LIHC and ZBTB34 expression are positively correlated. Our research provides further evidence that ZBTB34 is highly expressed in LIHC tissue. Combining the results of experiments and bioinformatics analysis, it is obvious that ZBTB34 is an oncogene that plays an important role in the development of LIHC.

The controversial expression pattern for various cancer types indicates ZBTB34 has a complicated role in the development of cancer. Although there is currently little research on the mechanism of ZBTB34 in cancer, extensive data have found that ZFPs play crucial roles in the development of cancer. Zinc finger motifs are categorized into several types, namely C2H2, C2HC, C2HC5, C2C2, CCCH, C3HC4, C4, C4HC3, C6, and C8 based on the position as well as the number of histidine and cysteine residues [24]. Because of the diversity of ZnF motifs, ZFPs coordinate a plethora of biological processes, including cell differentiation, cell apoptosis, and cell migration [25]. Over the last few decades, increasing evidence reveals that ZFPs play a role in the progression of liver cancer. For instance, the overexpression of ZNF143 facilitated HCC cell cycle progression via activating CDC6 [26]. ZNF384 could result in sluggish of the G1/S phase transition in HCC [27]. ZNF307 was reported to function as tumor suppressor in HCC [28]. ZNF263 could promote resistance to apoptosis in HCC [29]. ZNF689 may be a novel predictor for prognosis of patients with HCC [30]. Knockdown of ZNF233 suppresses HCC cell proliferation and tumorigenesis [31]. These mechanisms are related to ZFPs as transcription factors, regulating the expression of a number of genes with different functions, and these genes play important roles in the occurrence, development, recurrence, therapy resistance, and metastasis of HCC.

By conducting experiments and using bioinformatics analysis, we show that miR-125b-5p regulates the expression of ZBTB34. ZBTB34 expression has been shown to be upregulated in reactive oxygen species-induced apoptosis [32]. In recent work, we discovered that miR-125b-5p protects against apoptosis in endothelial cells under oxidative stress [33]. These results demonstrate that ZBTB34 and miR-125b-5p are both involved in oxidative stress reactions. The expression of three genes, ZBTB10, POLR1B, and AUH, was shown to be affected by ZBTB34. This finding suggests that there is a miR-125b-5p/ZBTB34/ (ZBTB10, POLR1B, AUH) signaling axis in LIHC. miR-125b-5p was found to be a tumor suppressor in HCC [34]. A previous study demonstrated that miR-125b-5p could be used as novel non-invasive biomarkers of HBV-positive HCC [35]. Furthermore, our study found that ZBTB34 could regulate the expression of ZBTB10, POLR1B, and AUH. A recent study discovered that ZBTB10 specifically interacts with the double-stranded telomeric variant repeat sequence TTGGGG by employing its tandem C2H2 zinc fingers [36]. Our earlier studies have demonstrated that ZBTB34 can bind to telomere DNA and lengthen telomeres [9]. In another study, it was discovered that oxidative stress regulates the expression of ZBTB10 and ZBTB34 [37]. These findings suggest that ZBTB34 and ZBTB10 are involved in oxidative stress reactions and have a binding relationship with telomeres DNA. POLR1B is the human Rpa2 homologue. It increases in rRNA synthesis that is associated with poor prognosis in patients with non-small cell lung cancer [38]. AUH gene encodes for a bifunctional mitochondrial protein with RNA-binding and hydratase activities. It has been discovered that AUH play a negative role in the development of HCC [39]. The relationship between ZBTB10, POLR1B, AUH, and ZBTB34 requires further investigation.

NLSs expose on the surface of a protein, and thus assist the protein in recognizing and binding to nuclear transport proteins [40]. By the online tool NLStradamus, a putative NLS at position ^320^RGGRARQKRA^329^ was predicted. In order to identify the functional NLS, we constructed the plasmid pEGFP-ZBTB34 and a series of mutations. The findings prove that the dysfunction of this NLS prevents the nuclear import of ZBTB34 in transfected cells, verifying this putative NLS is a functional monopartite NLS. NLS sequences could be varied with abundant basic residues, the arginine (R) or lysine (K) [41]. A monopartite NLS typically consists of a helix-breaking N-terminal proline or glycine followed by 7 to 11 basic residues with 4-8 basic amino acids, arginine or lysine [42]. For example, SV40 large T NLS (PKKKRKV) contains continuous 5 R/K residues whereas c-myc NLS (AAKRVKLD) has disrupted 3 R/K residues [43]. The monopartite NLS can be classified into “class 1-5” [44]. “Class 1” contains at least four consecutive basic amino acids, KR (K/R) R or K (K/R) RK. “Class 2” contains three basic amino acids and is represented by (P/R) XXKR (K/R). “Class 3”, “Class 4”, and “Class 5” are represented by KRX (W/F/Y) XXAF, (R/P) XXKR (K/R) and LGKR (K/R) (W/F/Y), respectively. The NLS within ZBTB34, however, does not match any consensus sequences of “class 1-5”, indicating that this NLS is a novel NLS. Mutations of the residues Lys^327^ and Arg^328^ resulted in ZBTB34 abolish in the nucleus, showing that these basic residues were critical determinants for the nuclear localization of ZBTB34.

**Figure 7:**
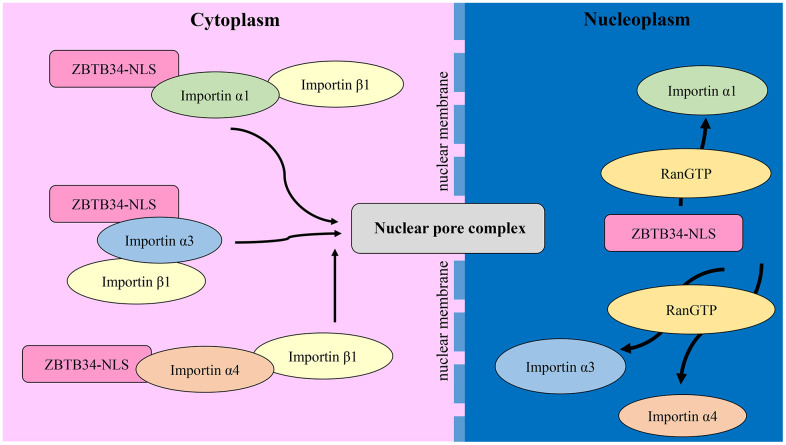
**The mechanism of ZBTB34 entering the nucleoplasm.** ZBTB34 binds to importins α1, α3, and α4 in the cytoplasm via its NLS and then recruits importin β1 to form a complex. These complexes transport ZBTB34 into nucleoplasm and then the importins are disassembled upon interaction with RanGTP.

Here, we demonstrate that the nuclear import of ZBTB34 is mediated by the importin α/β1 transport pathway. Through the NLS, the cargo protein binds to the nuclear import receptor proteins called importins. In mammalian cells, seven members of the importin family have been identified: importin α1, importin α3, importin α4, importin α5, importin α6, importin α7, and importin α8 [45]. Importin α isoforms are highly conserved, with 26% identity and 42% conservation in their amino acid sequences [46]. Importin α has three key structural domains: an N-terminal importin β-binding domain, armadillo repeats that function as internal cargo cNLS-binding sites, and a C-terminal region that binds to the nuclear export factor of importin α [47]. Importin α6 and α8 were omitted in our experiment since these two members were found to be testis or embryo-specific [48]. Using a co-immunoprecipitation assay, we demonstrated the roles of the adaptor proteins importin α1, α3, and α4 in the nuclear import of ZBTB34. The mechanism is that ZBTB34 binds to importin α1, importin α3, and importin α4. As seen in Figure 7, these importins α link ZBTB34 to importin β1, which then binds to nuclear pore complex proteins to import such complexes into the nucleoplasm, where they disassemble upon interaction with RanGTP [49].

In conclusion, our study demonstrated that the highly expressed ZBTB34 is associated with LIHC, and a putative miR-125b-5p/ZBTB34/ (ZBTB10, POLR1B, and AUH) signaling axis may be involved in the development of LICH. We also discovered a monopartite NLS (^320^RGGRARQKRA^329^) within ZBTB34. Lys^327^ and Arg^328^, two amino acids, are necessary for the import of ZBTB34 into the nucleus by mediating importin α1, α3, α4, and importin β1.

## MATERIALS AND METHODS

### Gene expression analysis

ZBTB34 was inputted in the TIMER2 web (http://timer.cistrome.org/). The expression difference of ZBTB34 between the tumor and adjacent normal tissues across different tumors was observed. Additionally, we used the “Pathological Stage Plot” module of the GEPIA2 web server (http://gepia2.cancer-pku.cn/#analysis) to obtain violin plots of the ZBTB34 expression in different pathological stages (stage I, stage II, stage III, and stage IV). The log2 [TPM (Transcripts per million) +1] transformed expression data were applied for the box or violin plots.

### Survival prognosis analysis

Overall survival significance map data of ZBTB34 across all TCGA tumors were obtained from the “Survival Map” module of GEPIA2. Cutoff-high (50%) and cutoff-low (50%) values were used as the expression thresholds for splitting the high-expression and low-expression cohorts. The log-rank test was used in the hypothesis test, and the survival plots of different tumors were also obtained through the “Survival Analysis” module of GEPIA2.

### Real-time PCR

The total RNA of cells and tissues from Chinese HCC patients was isolated using RNAzol (Sigma-Aldrich, China). mRNA was reversely-transcribed to cDNA using RevertAid first-strand cDNA synthesis kits (Sangon Biotech, China). qPCR analysis was conducted using primers 0.5 μL (Supplementary Table 1), cDNA template 2 μL, 2×SYBR Green PCR master mix 10 μL and dH_2_O 7 μL. The thermocycling program was 39 cycles of denaturation at 95° C for 15s, 55° C for 15s, with an initial cycle of 95° C for 3 min. The ago-miR-125b-5p and antago-miR-125b-5p have the same design as we previously reported [33]. The small nuclear RNA U6 and GAPDH were used as internal control. Relative expression values were calculated by the 2^-ΔCt^ method. All qPCR were performed at least three times.

### NLS predication

The online tool NLStradamus (http://www.moseslab.csb.utoronto.ca/NLStradamus/) was used to predicate the NLS sequences within ZBTB34. The three-dimensional structure of NLS was predicted by AlphaFold proteins structure database (https://alphafold.ebi.ac.uk/). Conservation analyses among multiple diverse species were employed by the NCBI Alignment Search Tool (https://blast.ncbi.nlm.nih.gov/Blast.cgi). The positions of ZnFs within ZBTB34 were predicted using the batch PWM software (http://zf.princeton.edu/index.php). The design of the ZBTB34-siRNA is same as we reported before through siDirect version 2.0 (http://sidirect2.rnai.jp/) [9].

### Human ZBTB34 cloning

ZBTB34 cDNAs were amplified by PCR using Pfu Turbo DNA polymerase (Agilent, China) and appropriate primers (Supplementary Table 1). After the digestion of PCR products with Xho I and BamH I, the cDNA fragment was inserted into the Xho I/BamH I sites of pEGFP-N1 to generate EGFP-ZBTB34 and EGFP-NLS fusion protein.

### Cell culture and transfection

HepG2 (No.CL-0103) cells were purchased from Procell Science Technology Co. Ltd, China and cultured in DMEM medium supplemented with 10% fetal bovine serum at 37° C in 5% CO_2_. The identity of HepG2 and its derivatives was confirmed by STR profiling. The cells were transfected with plasmid constructs (4.0 μg) using Lipofectamine 3000 (Invitrogen, China) and were stained with Hoechst 33342 (10 mg/ml). The fluorescence was visualized using an Olympus (BX51) fluorescent microscope.

### Alanine scanning technique

The NLS_A_ (Ala substitution of the NLS), NLS_M1_ (Ala substitution of Arg^320^), NLS_M2_ (Ala substitution of Arg^323^), NLS_M3_ (Ala substitution of Arg^325^), NLS_M4_ (Ala substitution of Lys^327^), and NLS_M5_ (Ala substitution of Arg^328^) were constructed using Site-Directed Mutagenesis Kit (Sangon Biotech, China). Details of primers used in the study are listed in Supplementary Table 1. The full-length sequence and all the mutants were confirmed by DNA sequencing.

### Co-immunoprecipitation (Co-IP) experiment

HepG2 cells were rinsed with ice-cold PBS and lysed in a lysis buffer containing proteinase inhibitors. Supernatants were collected, and protein concentrations were determined using Bradford Protein Assay Kit (Beyotime, China). Cells were co-transfected with EGFP-tagged expression plasmids for 24 hours and then collected and lysed on ice with 1 ml of lysis buffer. The lysate was subsequently incubated with an anti-EGFP monoclonal antibody (Sangon Biotech, China) and a 1:1 slurry of Protein G/A agarose beads (Millipore, China) overnight at 4° C. Then, lysis buffer was used to wash the beads three times. Finally, cell lysates were subjected to Western blotting analysis with anti-importin and anti-importin β1 antibodies.

### Western blotting

Cells were lysed for 1 hour on ice with NP-40 lysis buffer. Protein samples were separated by 10% SDS-PAGE. After electrophoresis, the separated proteins were transferred to a 0.45 μm nitrocellulose membrane. The membranes were blocked with 5% fat-free milk in PBS buffer for 1 hour at room temperature. After washing three times with PBS containing 0.1% Triton X-100, the membranes were incubated with antibodies in PBS with 0.1% BSA at 4° C overnight. Then, it was hybridized with horseradish peroxidase-conjugated secondary antibodies. Signals were developed by using an efficient chemiluminescence kit Enhanced (Proandy, Xi’an, China). The anti-GFP (No. D191040), anti-α-tubulin (No. D191049), anti-importin α1 (No. D194828), anti-importin α4 (No. D222548), anti-importin α5 (No. D197299), anti-importin α7 (No. D161794), anti-importin β1 (No. D197274) were purchased from Sangon Biotech, Shanghai, China. The anti-importin α3 (No. MABS1326) was purchased from Sigma-Aldrich, China.

### Statistical analysis

The data were expressed by mean ± SD. Differences were considered statistically significant at *P*<0.05. All statistical analyses were performed using SPSS version 18.0 (SPSS Inc., Chicago). All experiments were repeated 3 independent times.

## Supplementary Material

Supplementary Table 1
