# Supplementary material for: ZBTB34 is a hepatocellular carcinoma-associated protein with a monopartite nuclear localization signal
Source: Aging (Albany NY). 2023 Aug 30;15(16):8487–500. doi: 10.18632/aging.204987 (PMC10496988; doi:10.18632/aging.204987)
Supplement: Supplementary Table 1 [file aging-15-204987-s001.pdf]

SUPPLEMENTARY TABLE

Supplementary Table 1. The primers used in this study.

Primers	Sequences (5'→3')	Purpose
ZBTB34 cDNA	Forward: CTCGAG ATGGAAAGCACCCTGG	ZBTB34
	Reverse: GAGCTCTTAGTCAGGCGCATC	ZBTB34
NLS_A	Forward: CTTCGCTGCTGCTGCTGCTGCTGCTGCTGCTGCTCTGTC	NLS
	Reverse: AGCAGCAGCAGCAGCAGCAGCAGCAGCAGCGAAGCAGC	NLS
NLS_{M1}	Forward: GCTGCTTCAGCGGCCGTGG	R334
	Reverse: GCTGAAGCAGCTCAGC	R334
NLS_{M2}	Forward: CGAGGCGCTGGGGCCCGCCAG	R336
	Reverse: AGCGCCTCGGAAGCAGCTC	R336
NLS_{M3}	Forward: GGCCGCTCAGAAGCGAGC	R339
	Reverse: AGCGGCCCCACGGCCTCG	R339
NLS_{M4}	Forward: CCCGCCAGGCTCGAGCTCTGTCTG	K341
	Reverse: AGCCTGGCGGGCCCCACGG	K341
NLS_{M5}	Forward: CCAGAAGCGAGCTCTGTCTGTTC	R342
	Reverse: AGCCTTCTGGCGGGCCCCACGG	R342
siRNA 1	Guide: UUCACUAAGCUAAAUUCAGCA	ZBTB34
	Passenger: CUGAAUUUAGCUUAGUGAAGG	ZBTB34
siRNA 2	Guide: AACAGAAAAUAACCAUUAGAA	ZBTB34
	Passenger: CUAAUGGUUAUUUUCUGUUGA	ZBTB34
miR-125b-5p-F	ACACTCCAGCTGGGTCCCTGAGACCCTAACT	qPCR
miRNA-125b-5p-R	CTCAACTGGTGTCGTGGA	qPCR
U6-F	CTCGCTTCGGCAGCACA	qPCR
U6-R	AACGCTTCACGAATTTGCGT	qPCR
MiR125-125b-5p siRNA	AACCTGAAGATCTTCAACAACCCTGTCTC	Ago-mir
MiR125-125b-5p siRNA	AAGTTGTTGAAGATCTTCAGGCCTGTCTC	Ago-mir
MiR125-125b-5p siRNA	TCTACCTTGCCTTCAGATTCATAAT	Antago-mir
MiR125-125b-5p siRNA	TGACTCTTGGACTTGTCATCCTCCC	Antago-mir
ZBTB10-F	CGGCTCCACGAACAATAACG	qPCR
ZBTB10-F	CAGGCCCTCCAATTCCACTT	qPCR
POLR1B-F	TCCGAATGTTGATTATGCCTCG	qPCR
POLR1B-R	TGACAGCGGAATGTTCTTCCC	qPCR
AUH-F	ATTGGCGATTATTCCTGGTGG	qPCR
AUH-R	GAACGTGGCTGATTAAGCCCA	qPCR
CDKN1A-F	TGTCCGTCAGAACCCATGC	qPCR
CDKN1A-R	AAAGTCGAAGTTCCATCGCTC	qPCR
MYLIP-F	GCAGGCGACTGGGAATCATAG	qPCR
MYLIP-R	CGGTTTCTCAGGTTTAGCCAT	qPCR
EHMT1-F	CATGCAGCCAGTAAAGATCCC	qPCR
EHMT1-R	CTGCTGTCGTCCAAAGTCAG	qPCR
HMGB4-F	TCGACAAAGCCCGATACCAG	qPCR
HMGB4-R	TCTTGGCAGAAGAGTAGGAAGG	qPCR
LGALS4-F	CGACGCTGCCTTACTACCAG	qPCR
LGALS4-R	CCAACCACAAAGTTCACGAAGA	qPCR
GAPDH-F	GCACCGTCAAGGCTGAGAAC	Control
GAPDH-R	TGGTGAAGACGCCAGTGGA	Control
